# Research Progress on Seneca Valley Virus Infection in Swine: A Review

**DOI:** 10.3390/vetsci13101106

**Published:** 2026-10-17

**Authors:** Zhiqiang Hu, Lili Wu, Ran Guan, Chaoyun Yang, Xi Chen, Zengwen Huang, Guangwen Yan, Guiying Hao

**Affiliations:** 1 College of Animal Science, Xichang University, Xichang 615013, China; 2 Key Laboratory of Animal Epidemic Disease Detection and Prevention in Panxi District, Xichang University, Xichang 615013, China; 3 Liangshan Ecological Breeding Engineering Technology Research Center, Xichang University, Xichang 615013, China; 4 College of Veterinary Medicine, Northeast Agricultural University, Harbin 150030, China; 5 Shandong New Hope Liuhe Group Co., Ltd., Qingdao 266000, China

**Keywords:** Seneca Valley virus, virological characteristics, epidemiology, transmission, host-virus interaction, innate immunity, diagnosis

## Abstract

Seneca Valley virus (SVV), also referred to as Senecavirus A (SVA), is an emerging pathogen within the *Picornaviridae* family. Since its initial identification, SVV has been implicated in the onset of porcine idiopathic vesicular disease (PIVD) across several prominent pig-producing nations globally. Due to the clinical manifestations of SVV being challenging to differentiate from those of significant animal diseases such as foot-and-mouth disease (FMD), it poses considerable economic burdens and biosecurity challenges to the swine industry. This review endeavors to provide a comprehensive synthesis of the current state of research on SVV, systematically detailing its virological characteristics, molecular biology, global epidemiology, and evolutionary dynamics. The focus is placed on elucidating its pathogenic mechanisms in swine and the intricate molecular networks that govern its interactions with the host. Particular attention is given to the strategies employed by viral proteins to counteract host innate immune responses and to modulate autophagy, apoptosis, and other critical signaling pathways. Furthermore, we consolidate the existing diagnostic technologies and advancements in vaccine development, and we explore prospective research directions, aiming to furnish a reference for the clinical prevention and control of SVV.

## Simple Summary

Seneca Valley virus (SVV) represents an emerging pathogen affecting swine, characterized by a vesicular disease that closely mimics foot-and-mouth disease (FMD), a highly concerning ailment in livestock. This phenotypic similarity complicates accurate diagnosis and imposes significant challenges on global swine production. This review synthesizes current knowledge regarding SVV, beginning with an overview of its fundamental characteristics, geographical dissemination, and evolutionary dynamics. Subsequently, we delve into the pathogenesis of SVV in swine, emphasizing the mechanisms by which viral proteins manipulate cellular autophagy and apoptosis, as well as their role in evading porcine immune responses, particularly the interferon pathway. Furthermore, we evaluate the diagnostic methodologies available for SVV detection and discuss advancements in vaccine development. By integrating these insights, this review aims to enhance the capacity of veterinarians and researchers to effectively manage and mitigate SVV infections.

## 1. Introduction

Seneca Valley virus (SVV), also referred to as Senecavirus A (SVA), is a single-stranded positive-sense RNA virus classified under the genus Senecavirus within the family *Picornaviridae* [1,2]. The virus was initially isolated and identified in the United States in 2002 as a contaminant in cell culture [2,3]. Although SVV was not originally regarded as a significant animal pathogen, since 2014, it has been associated with outbreaks of porcine idiopathic vesicular disease (PIVD) in several major pig-producing countries, including Brazil, the United States, and China [2,4]. Infected pigs exhibit vesicles and ulcerations on the snout and coronary band, along with lameness, and the virus can result in acute mortality in newborn piglets, leading to substantial economic losses and biosecurity threats to the global swine industry [5,6]. The clinical manifestations of SVV are challenging to differentiate from those of major animal diseases requiring stringent control measures, such as foot-and-mouth disease (FMD), thereby complicating differential diagnosis and disease management efforts [7].

Epidemiologically, SVV is characterized by an expanding geographic distribution and growing genetic diversity. In China, since its initial identification in 2015, the virus has swiftly disseminated to most provinces across the country [8]. Periodic outbreaks have also been documented in other major swine-producing nations, including Brazil and the United States [9,10]. Phylogenetic analyses indicate that SVV has diverged into at least eight distinct genetic lineages, with circulating strains in various regions exhibiting unique evolutionary characteristics [11]. The rapid genetic divergence of the virus, coupled with the co-circulation of multiple lineages, complicates efforts in viral source tracing and disease prevention and control, while also presenting significant challenges to the broad protective efficacy of vaccines.

In recent years, research on SVV has progressed swiftly, encompassing various levels from fundamental virology to clinical applications. This review seeks to systematically synthesize and assess SVV in terms of its virological properties, global epidemiological trends, molecular pathogenic mechanisms, and host interactions. The objective is to furnish a comprehensive resource for researchers in the field and establish a theoretical foundation for the future prevention, control, and clinical utilization of SVV.

## 2. Virology and Molecular Biology

### 2.1. Genomic Structure and Protein Functions

**Figure 1:**
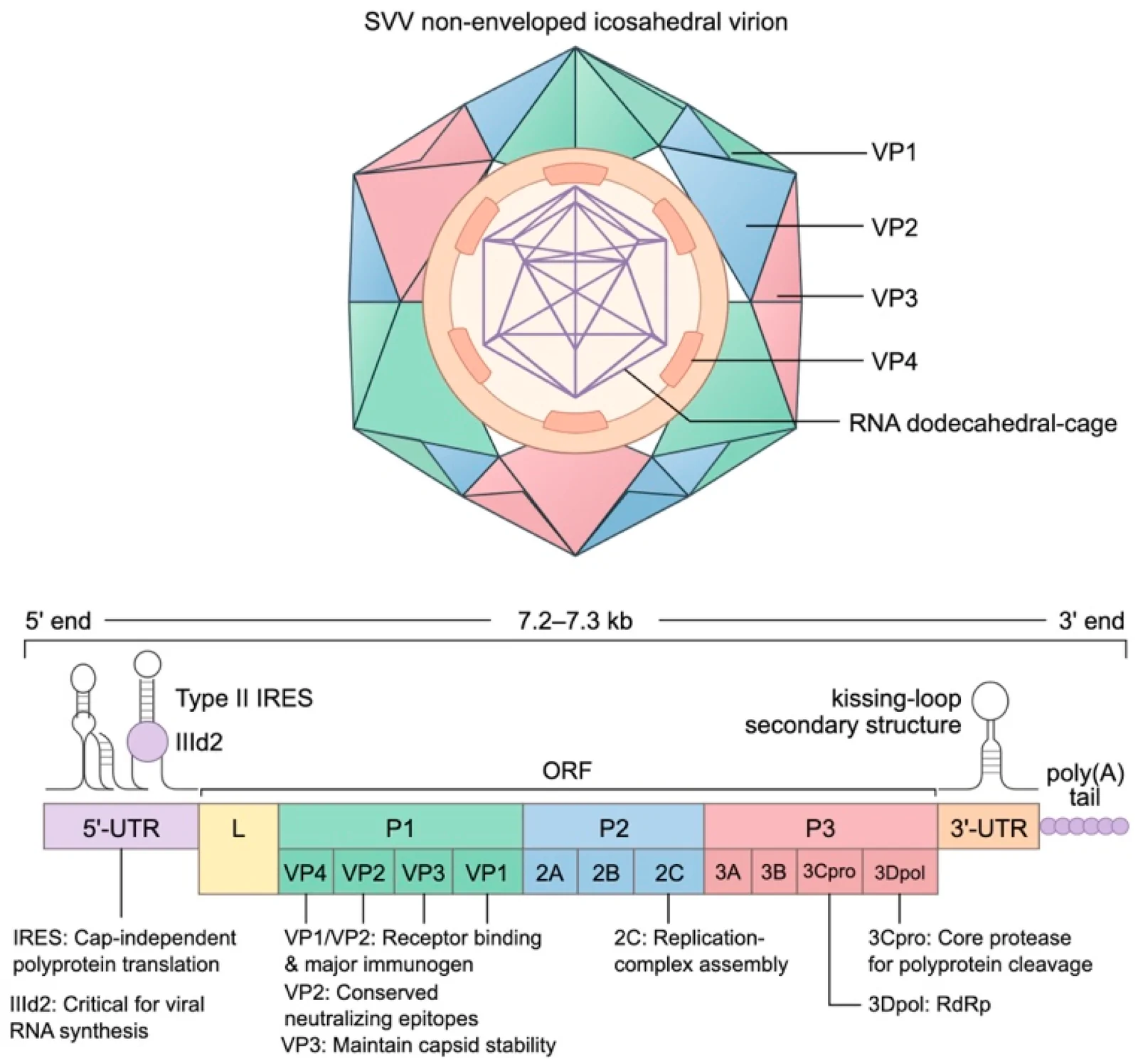
Genomic structure and protein functions of SVV (By Figdraw).

SVV represents the sole species within the genus *Senecavirus*, belonging to the family *Picornaviridae*. It is characterized as a non-enveloped, single-stranded positive-sense RNA virus [12]. The viral genome spans approximately 7.2 to 7.3 kb and exhibits the hallmark features of picornaviruses, including a large open reading frame (ORF) bordered by highly structured 5′ and 3′ untranslated regions (UTRs), as well as a polyA tail at the 3′ terminus (Figure 1) [13,14,15]. In mature virions, a portion of the genomic RNA forms a structured dodecahedral cage that closely associates with the inner surface of the capsid, thereby contributing to the stability of the virion [16]. The 5′ UTR of SVV harbors a type II internal ribosome entry site (IRES), structurally analogous to the IRES of hepatitis C virus (HCV) [17]. IRES elements are typically composed of multiple complex stem-loop domains and pseudoknot structures, facilitating the direct recruitment of ribosomes to initiate translation of the viral polyprotein independently of the host’s m7G cap structure [18]. Research has demonstrated that the pseudoknot structure within the SVV IRES is essential for its functional integrity [19]. However, the IIId2 subdomain serves a distinct role; while it is not indispensable for the translation-initiation activity facilitated by the SVV IRES, it is vital for efficient viral RNA synthesis and overall viral proliferation [20]. This is markedly different from Classical swine fever virus (CSFV), where IIId2 is crucial for ribosome assembly and IRES activity, suggesting that analogous RNA structures may have evolved to fulfill divergent functions in distantly related viruses [20]. Similarly, the 3’ UTR contains significant secondary structures, including stem-loops that form a kissing-loop, which are integral to viral replication and translational regulation [15]. The viral genome encodes a single polyprotein, organized into a leader protein (L) followed by three regions: P1, P2, and P3. Post-translationally, this polyprotein is cleaved by the virally encoded 3C protease (3Cpro), among others, to yield mature viral proteins, including four structural proteins (VP1, VP2, VP3, and VP4, encoded by the P1 region) and multiple nonstructural proteins (encoded by the P2 and P3 regions) [14,15].

The structural proteins VP1, VP2, and VP3 collectively constitute the external surface of the viral capsid, while VP4 is situated internally (Figure 1). These proteins fulfill multiple functions throughout the viral life cycle. The external loop regions of VP1 and VP2 serve as critical sites for binding to host cell receptors, facilitating viral attachment and entry [21]. Moreover, they represent the primary immunogenic components that elicit neutralizing antibodies in the host, with conserved neutralizing epitopes identified within VP2 [22,23]. The VP3 protein primarily plays a role in maintaining capsid stability. The VP4 protein, located inside the capsid, participates in the assembly and maturation of viral particles.

Nonstructural proteins are integral to viral replication (Figure 1). The 2C protein, a multifunctional entity, is primarily involved in viral RNA replication and the activation of the host DNA damage response (DDR) signaling pathway [24]. 3Cpro represents a central proteolytic enzyme in SVV, responsible for recognizing and processing most cleavage sites in the viral polyprotein precursor [25]. This activity underpins the maturation of both structural and non-structural proteins, positioning 3Cpro as an indispensable regulator of viral replication. The 3D polymerase (3Dpol), an RNA-dependent RNA polymerase (RdRp), is responsible for catalyzing the replication of the viral genome. The aforementioned viral proteins not only mediate viral replication and assembly but also exert regulatory effects on cellular processes and antagonize host immunity by targeting diverse host proteins and signaling pathways. The relevant pathogenic mechanisms are comprehensively reviewed in Section 5.

### 2.2. Replication Cycle of SVV

**Figure 2:**
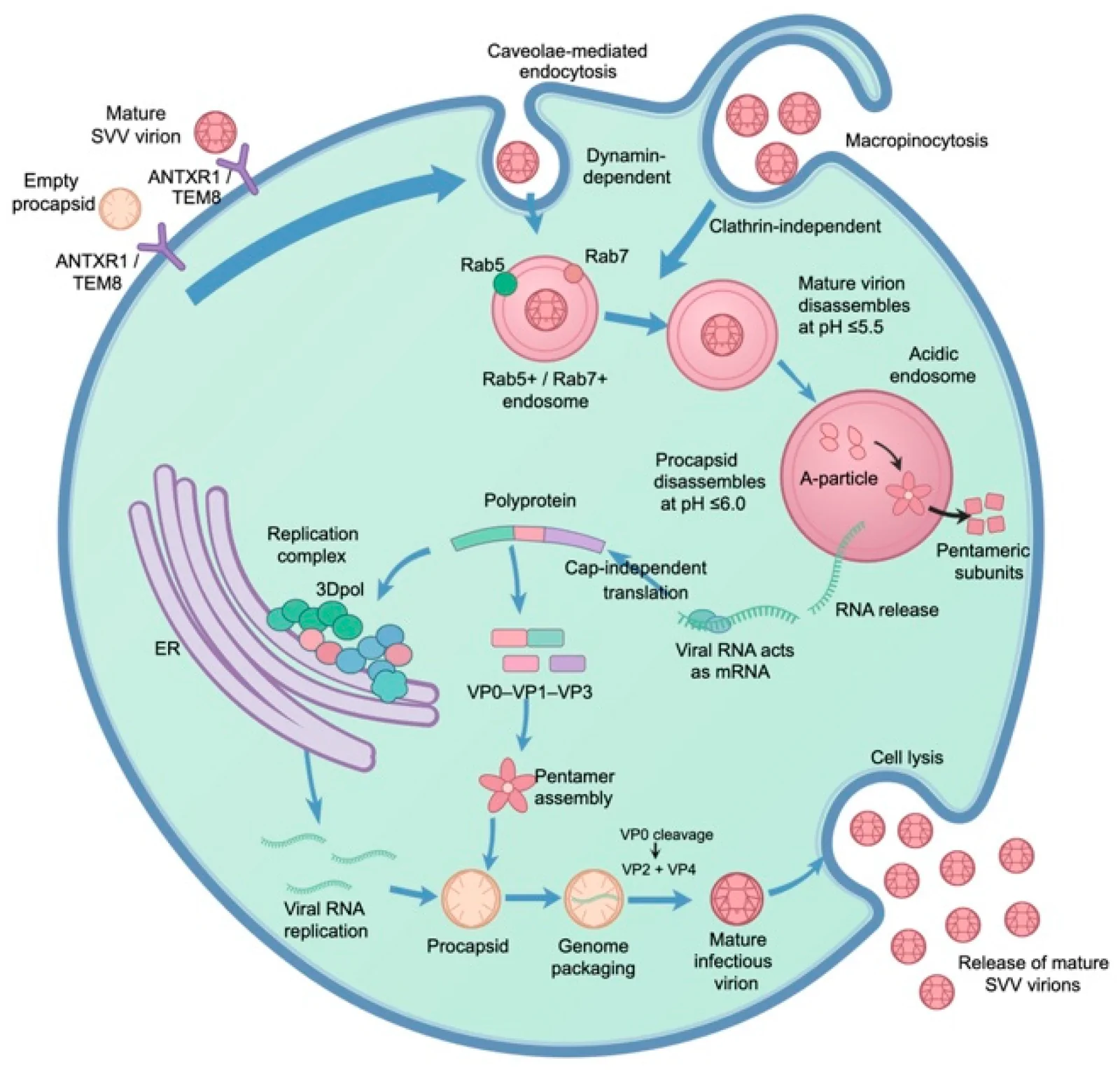
The replication cycle of SVV (By Figdraw).

The infection of host cells by SVV is a complex, multi-step process initiated by the binding of the virion to specific receptors on the cell surface (Figure 2). The primary receptor for SVV is anthrax toxin receptor 1 (ANTXR1), also referred to as tumor endothelial marker 8 (TEM8) [26,27]. The binding to ANTXR1 is facilitated by specific loop structures on the capsid proteins VP1 and VP2 [21]. Research has demonstrated that N-linked glycosylation of ANTXR1 is crucial for the efficient attachment and infection by SVV [28]. During viral replication, a significant number of naturally occurring empty capsids (procapsids), which possess an external structure identical to that of mature virions, are produced and can also efficiently bind to ANTXR1 [16]. Following receptor binding, the virus enters the cell through multiple endocytic pathways. In the porcine kidney cell line PK-15, SVV predominantly enters cells via caveolae-mediated endocytosis and macropinocytosis, a process that necessitates dynamin but is independent of clathrin-mediated endocytosis [16]. Upon entry, the virus becomes encapsulated within endosomes and is transported along the endo-lysosomal pathway, a process orchestrated by the small GTPases Rab5 and Rab7 [29]. Within the acidic milieu of the endosomes, the viral capsid undergoes a series of conformational transformations that culminate in uncoating and the release of the viral genome. Cryo-electron microscopy studies have demonstrated that acidic pH conditions induce the dissociation of the SVV capsid into pentameric subunits, thereby facilitating the release of the RNA genome into the cytoplasm. This process is hypothesized to involve a transient, conformationally altered “A-particle” intermediate [30]. Experimental data indicate that mature SVV virions exhibit greater resistance to acidic conditions compared to their empty capsids (procapsids); the former disassemble only at pH ≤ 5.5, whereas the latter commence dissociation at pH ≤ 6.0. This increased stability is believed to be imparted by the interaction between the structured RNA genome and the capsid proteins within the virion [16]. Once the viral RNA genome enters the cytoplasm, it immediately functions as mRNA, utilizing its IRES element to initiate the translation of the polyprotein. Simultaneously, the virus establishes replication complexes on host organelle membranes, notably the endoplasmic reticulum (ER) membrane, with 3Dpol serving as the central component of RNA replication. Infection with SVV induces ER stress, specifically activating the PERK and ATF6 signaling pathways, which promote viral replication and the induction of autophagy [31,32]. Newly synthesized viral RNA and structural proteins assemble into new virions within the cytoplasm. Initially, VP0, VP1, and VP3 assemble into pentamers, which subsequently self-assemble into procapsids [16]. The viral genome is then encapsulated within these structures. During or following encapsulation, the precursor protein VP0 is cleaved into VP2 and VP4, resulting in the formation of infectious mature virions. Ultimately, the accumulation of replicated virions leads to host cell lysis and subsequent release.

## 3. Epidemiology and Evolution

### 3.1. Global Distribution and Genetic Diversity

Since 2014, the SVV has been responsible for outbreaks of vesicular disease in several prominent pig-producing countries and regions, thereby posing a novel threat to the global swine industry [33]. A comprehensive systematic review and meta-analysis conducted globally, encompassing data from 2014 to 2020, indicated that the aggregated global prevalence of SVV is approximately 15.90%. This analysis also highlighted significant regional disparities, with a higher prevalence observed in the Americas compared to Asia. Additionally, it was noted that the global prevalence of SVV declined post-2016, potentially as a result of enhanced control measures implemented in the major affected countries [34]. The initial outbreaks were predominantly concentrated in Brazil, the United States, and China [35,36,37]. In Brazil, outbreaks have persisted since their initial report in 2015, exhibiting a pattern of periodic recurrence [9]. The interval between the first (2014–2015) and second (2018) waves was approximately three years, whereas the third wave, which commenced in May 2020 in the primary pig-producing regions, was separated from the preceding wave by only 1.5 years, suggesting a reduction in the intervals between outbreak waves [9]. In the United States, outbreaks also spread extensively within breeding herds, resulting in increased neonatal mortality and vesicular lesions in sows [10,35]. This aligns with epidemiological data, as the aforementioned meta-analysis identified newborn piglets as the highest-risk group, with an infection rate of up to 71.69% [34]. In China, following the initial detection of SVV in Guangdong Province in 2015, the virus rapidly disseminated across multiple provinces; by the end of 2019, more than half of the provinces, autonomous regions, and municipalities had reported SVV infections [8,37]. In recent years, the geographic distribution of SVV has further expanded, with the first outbreak documented in Chile in 2022 [38].

The extensive geographic spread is accompanied by significant genetic diversity. Phylogenetic analyses indicate that SVV has diverged into at least eight distinct genetic lineages [11]. Strains from different regions exhibit unique evolutionary characteristics. For instance, strains circulating in Brazil are genetically distinct from those in other countries and form an independent monophyletic clade, suggesting they may have originated from a single introduction event followed by localized, independent evolution [2,9]. Subsequent research has elucidated the dynamics of this local evolution: strains isolated in Brazil during 2018 and 2020 constitute a novel genetic cluster, distinct from the earlier 2015–2016 Brazilian strains, indicating that the emergence of new viral variants may contribute to recurrent outbreaks [36]. In contrast, the viral strains circulating within China can be categorized into at least five distinct genetic lineages. The scenario is marked by the concurrent circulation of multiple genetic clusters and a temporal progression of dominant strains [37,39]. Initially, the epidemic dynamics were primarily driven by domestic transmission; for instance, the HB-CH-2016 strain, isolated in Hubei in 2016, exhibits a close genetic relationship to the first Chinese isolate, CH-01-2015, from Guangdong [8]. Nevertheless, subsequent investigations have identified that strains emerging in China post-2017 bear a distant genetic relationship to earlier Chinese strains but show high homology with 2015 US isolates, such as USA/GBI29/2015 [40,41]. These findings elucidate a complex global transmission network for SVV, characterized by the virus’s continuous evolution and frequent long-distance dissemination across diverse geographic regions.

### 3.2. Viral Evolution and Recombination

As an RNA virus, SVV demonstrates a relatively high evolutionary rate, primarily driven by a high nucleotide substitution rate and purifying selection, which results in the rapid accumulation of genetic variation [11]. An analysis of strains circulating during 2015–2016 quantified this evolutionary rate at an average of 9.96 × 10^−3^ substitutions per site per year. This study also revealed that, in comparison to the 2002 prototype strain (SVV-001), these more recent strains had accumulated 25 fixed nonsynonymous mutations across eight mature viral proteins, including the capsid proteins [42]. Furthermore, in vitro serial-passage experiments have corroborated the rapid mutability of the SVV genome: over the course of 80 passages, 41 within-host single-nucleotide variants (SNVs) emerged in the viral progeny, with the majority resulting in nonsynonymous mutations [43].

In addition to point mutations, genetic recombination serves as a crucial mechanism contributing to the genetic diversity and adaptive evolution of SVV. Among three virus strains co-circulating on a single pig farm, one strain, designated as CH-GDSG-2018-3, was identified as a recombinant of two other Chinese circulating strains, CHhb17 and HeNKF-1, with recombination breakpoints predominantly located in the regions encoding the structural proteins VP1, VP2, and VP3. Notably, this recombinant strain demonstrated a greater proliferative capacity in vitro compared to the two non-recombinant viruses isolated from the same farm, thereby providing direct evidence that recombination events may confer a growth advantage to the virus [44]. Further analysis of strains circulating in Guangdong Province, China, in 2019, revealed a more intricate recombination pattern: one strain, CH-GDZS-2019, was identified as a recombinant of two 2015 US strains, while another strain, CH-GDMZ-2019, resulted from recombination between two local Chinese circulating strains [45]. Similarly, the analysis of the USA/GBI29/2015-like strain CH-GDZQ-2018, isolated in Guangdong in 2018, confirmed recombination among local strains [46]. This mechanism facilitates the rapid integration of genetic material from diverse viral lineages, potentially leading to the emergence of strains with novel characteristics, such as increased proliferative capacity, thereby complicating viral traceability and control efforts [44,45].

### 3.3. Transmission Routes and Host Range of SVV

**Figure 3:**
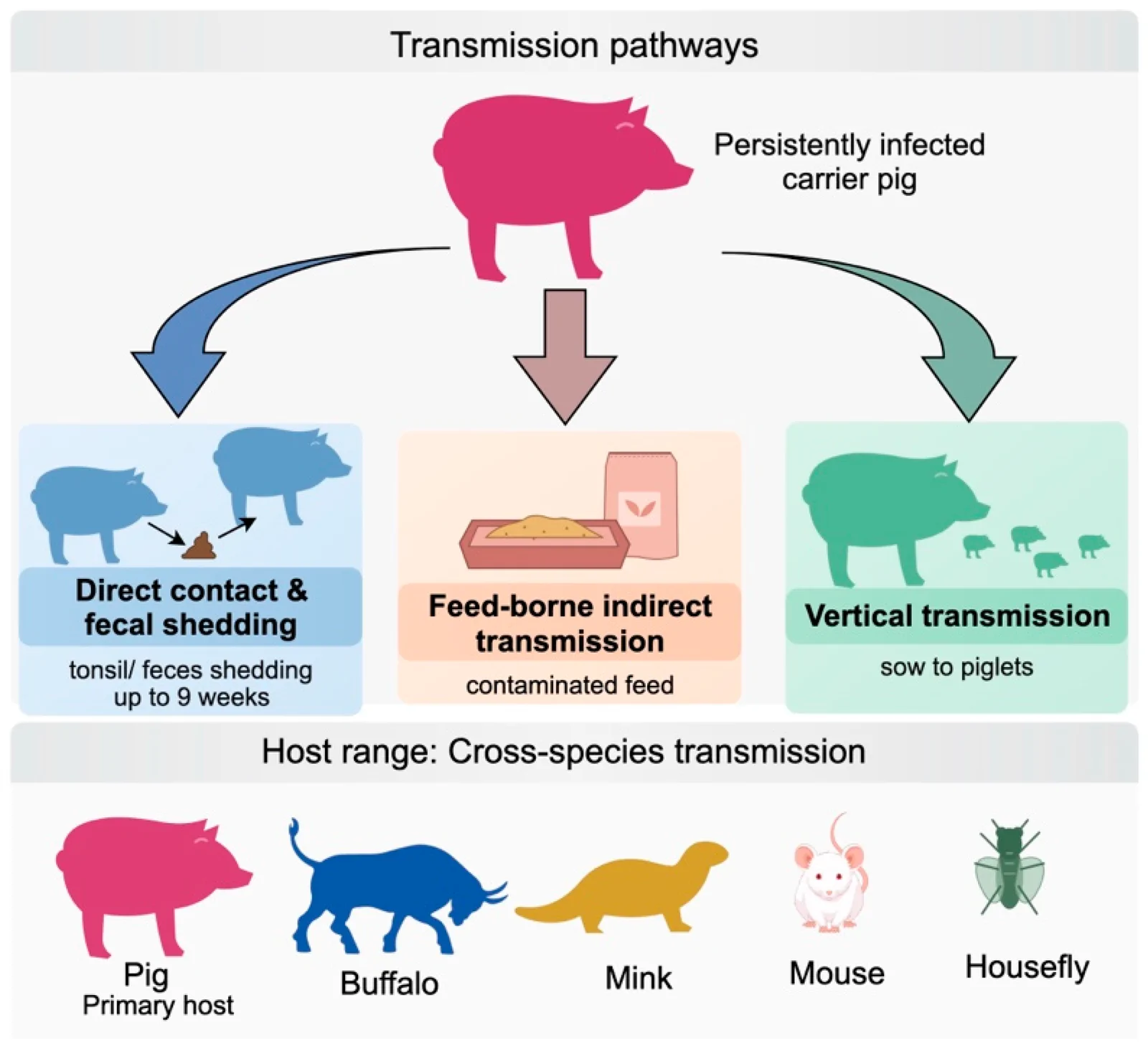
Transmission routes and host range of SVV (By Figdraw).

SVV is transmitted through both horizontal and vertical pathways, with persistent infections in carrier pigs playing a crucial role (Figure 3). While virus shedding in vesicular fluid and blood is transient, shedding in the tonsils and feces can continue for up to nine weeks [47]. SVV can establish a persistent infection, with viral RNA remaining in lymphoid tissues for up to 60 days and intermittent shedding occurring [48]. Asymptomatic carrier pigs represent a potential infection source [36], and subclinically infected piglets can disseminate the virus over long distances prior to symptom onset [47]. Contaminated feed also acts as a vehicle for indirect transmission [49]. Furthermore, experimental infection of pregnant sows has provided evidence for vertical transmission occurring both in utero and perinatally. Transplacental infection was inferred from viremia and seropositivity detected in piglets before colostrum ingestion, given that maternal antibodies cannot cross the porcine epitheliochorial placenta [6]. Piglets born to persistently infected sows also shed virus in oro-rectal secretions and harbor viral RNA in the tonsil, indicating efficient perinatal transmission through contact with shedding dams [48]. Notably, experimentally infected neonates remained clinically normal, and challenge did not reproduce the neonatal mortality reported in the field.

The host range of SVV is broadening, facilitating cross-species transmission (Figure 3). Beyond pigs, the virus has been identified in mice and houseflies [50]. Strains transmitted from pigs have been isolated from symptomatic buffalo, demonstrating a wide cell tropism [51]. Experimental and serological investigations have further validated that SVV can infect mink, leading to disease manifestation and viral shedding [50].

## 4. Clinical Signs and Pathology in Pigs

SVV infection can induce clinical disease across various age groups in swine. In sows and finishing pigs, the primary clinical manifestations include vesicular lesions, ulceration, and lameness affecting the feet, as well as areas surrounding the mouth and nose. These symptoms are often indistinguishable from those of FMD [52], although it is noteworthy that foot lesions may present several days prior to the appearance of oral and nasal lesions [53]. Conversely, in neonatal piglets, SVV infection is typically fatal, characterized by high mortality rates, lethargy, diarrhea, and neurological symptoms [6,35,54]. In nursery pigs, the infection results in milder clinical signs, such as transient low-grade fever and vesicular lesions, with viremia that can persist for over 14 days [55,56]. Experimental studies indicate that viremia is detectable as early as the third day post-infection, and seroconversion is observed in all pigs by the fifteenth day [53]. The pathogenicity of SVV strains varies, with recent strains exhibiting greater pathogenicity compared to historical strains [57,58,59,60]. Nevertheless, a comprehensive and systematic quantitative comparison of pathogenicity across its various genetic lineages is still absent. Current pathogenicity assessments have predominantly been limited to pairwise comparisons, such as those between historical and contemporary strains [57,58], two Chinese strains [59], or two distinct phylogenetic branches [61]. These studies have not provided a lineage-wide quantitative analysis. This deficiency can be attributed to several factors, including the recent establishment of a consolidated SVV lineage classification, the lack of a standardized animal challenge model, and the use of variable virus doses and inoculation routes. Additionally, the absence of unified clinical and pathological scoring systems further complicates direct cross-lineage comparisons of virulence. Pathogenicity is significantly influenced by the age of the host, as demonstrated by findings that pigs aged 30–65 days showed no clinical signs when challenged with SVV-CH-SD, whereas pigs aged 90–100 days developed vesicular lesions and lameness [14]. Furthermore, the genetic background of the host [62] and stress status [48] are critical factors influencing the severity of the disease.

The histopathological analysis demonstrates that SVV induces multi-organ damage. Characteristic pathological alterations in the skin and mucosa include vacuolar degeneration of epithelial cells, which leads to vesicle formation [52]. Microscopically, epidermal vesicles and infiltration of inflammatory cells are evident [56]. In pigs succumbing to the infection, multisystemic lesions are observed, such as nonsuppurative myocarditis, encephalitis, interstitial pneumonia, and atrophy of intestinal villi [63]. Notably, lymphoid tissue damage is significant. SVV induces immunosuppression by selectively promoting apoptosis of B lymphocytes, potentially contributing to the high mortality rate observed in neonatal piglets [64]. The virus is distributed extensively across various organs, with a particularly high concentration in the tonsils and inguinal lymph nodes [55,56]. SVV is capable of establishing long-term persistent infections, with the tonsils serving as a critical site for viral persistence [48], and lymphoid tissues acting as a major reservoir [60]. The virus can be detected in serum, nasal swabs, fecal swabs, oral fluid, and semen [35,56,60].

## 5. Pathogenesis and Host-Virus Interaction

### 5.1. Regulation of Cellular Processes by SVV

#### 5.1.1. SVV and Cellular Autophagy

As illustrated in Table 1, SVV infection induces a complete autophagic flux, exhibiting species-specific effects: it enhances viral proliferation in porcine cells while suppressing it in human cell lines [65]. In porcine cells, autophagosomes function as sites for viral replication [32]. The initiation of autophagy is intricately linked to ER stress, specifically through the PERK and ATF6 arms of the unfolded protein response (UPR) [32]. The virus exploits this mechanism to specifically degrade STING via reticulophagy, thereby facilitating immune evasion [66]. The viral proteins VP1, VP3, and 3C collaboratively induce autophagy by activating the AKT-AMPK-MAPK signaling pathway and inhibiting the downstream mTOR pathway [67]. To evade clearance by defensive autophagy, SVV 3Cpro cleaves the selective autophagy receptor OPTN at Q513 [68], and another restriction factor, SQSTM1/p62, at E355, Q392, and Q395, thereby nullifying its antiviral activity [65]. Additionally, the viral protein VP1 interacts with SQSTM1/p62, promoting its degradation [67]. Furthermore, the virus employs autophagy to degrade the antiviral protein cGAS [69] or disrupts mTOR signaling by cleaving EphA2, thereby further modulating autophagy [70].

#### 5.1.2. SVV and Programmed Cell Death

As illustrated in Table 1, SVV initiates apoptosis at a later stage of infection to facilitate efficient viral release and dissemination [71]. The viral protease 3Cpro is essential for the activation of host caspase-3, -8, and -9 signaling pathways [71,72]. Virus-induced ER stress serves as an upstream event in the activation of mitochondrial apoptosis, resulting in mitochondrial Ca^2+^ overload via the PERK signaling pathway [31]. Notably, SVV is among the few RNA viruses capable of inducing pyroptosis, primarily due to the direct cleavage of porcine gasdermin D (GSDMD) by 3Cpro at Q193 and Q277 [72,73]. Furthermore, recent findings indicate that 3Cpro actively suppresses GSDMA-mediated pyroptosis by cleaving gasdermin A (GSDMA) at Q187-G188 [74]. Additionally, 3Cpro concurrently cleaves NLRP3 at Q305 to inhibit inflammasome activation [72,75]. In contrast to the suppressive role of 3Cpro, 3Dpol serves as a potent activator of the NLRP3 inflammasome [76], suggesting an antagonistic regulatory mechanism between distinct viral proteins. Additionally, SVV can induce ferroptosis through a mechanism involving the degradation of GPX4 by 3Cpro and the facilitation of NCOA4-mediated ferritinophagy by 3Dpol. These processes collectively result in the accumulation of lipid peroxides, ultimately triggering ferroptosis [77].

#### 5.1.3. SVV and Other Cellular Processes

As illustrated in Table 1, SVV infection induces DNA double-strand breaks (DSBs) in the host and activates the DNA damage response (DDR); however, the virus employs a “damage without repair” strategy. The viral nonstructural proteins 2B and 2C play pivotal roles in activating the DDR while simultaneously inhibiting DNA repair. These proteins achieve this by disrupting the condensation of 53BP1 at DSB sites, thereby creating an environment conducive to viral replication [24]. Additionally, SVV modulates host cellular translation processes: the viral protease 3Cpro inhibits host protein synthesis by cleaving PABPC1 [78], while the virus recruits the ribosomal protein RPL13 to enhance its IRES-driven translation [18]. Furthermore, SVV dynamically influences stress granule (SG) formation. During the early stages of infection, SVV transiently induces SGs via the PKR-eIF2α signaling pathway; in the later stages, 3Cpro disassembles SGs by disrupting the G3BP1-eIF4G1 interaction or cleaving eIF4G1, thereby ensuring efficient translation of viral proteins [72,79]. In terms of cellular metabolism, SVV elevates aspartate levels by upregulating the amino acid transporter SLC38A8 to facilitate its replication [80]. Additionally, SVV infection perturbs host copper homeostasis, resulting in copper accumulation that subsequently inhibits the NF-κB signaling pathway, thereby attenuating innate immune responses [81].

### 5.2. Interaction Between Host Innate Immunity and SVV

#### 5.2.1. Host Innate Immune Recognition and Response to SVV

Host cells primarily detect SVV through the retinoic acid-inducible gene I-like receptor (RLR) and cGAS-STING pathways, with RIG-I serving as the principal sensor that identifies SVV RNA and initiates downstream antiviral signaling, thereby playing a pivotal role in limiting viral replication [82]. Transcriptomic analyses corroborate the activation of the RIG-I, NF-κB, and JAK-STAT signaling pathways following SVV infection [83]. The mitochondrial protein Mfn2 functions as a negative regulator of RIG-I signaling; its downregulation by the virus alleviates the inhibition of the RIG-I/IRF7 signal, thereby enhancing the production of type III interferon IFN-λ3 [75]. SVV infection also induces mitochondrial dysfunction, leading to the release of mitochondrial DNA (mtDNA) into the cytoplasm, where it is detected by the cGAS-STING pathway, subsequently activating type I interferon production [69]. Consequently, STING agonists have been shown to effectively activate the STING signaling pathway in porcine cells, thereby inhibiting SVV replication [84]. Additionally, the TLR pathway is involved in viral recognition; however, its activation is modulated through the virus-downregulated lncRNA 8244-miR-320-CCR7 axis [85]. The activity of host innate immune cells is essential for controlling SVV infection and varies significantly between host species [62]. Among the activated interferon-stimulated genes (ISGs), the product of CH25H, 25-hydroxycholesterol (25HC), demonstrates potent suppression of SVV by inhibiting viral adsorption [86]. While viral recognition elicits a robust proinflammatory response, the expression of IL-1α/β and TNF-α promotes viral replication, in contrast to the chemokines CCL2, CCL5, and CXCL10, which exhibit antiviral properties [87]. Furthermore, the virus enhances inflammation by persistently activating the DDR-ATM-NF-κB pathway [24], while simultaneously counteracting NF-κB signaling through the disruption of copper homeostasis [81]. Viral proteins exhibit complex functionalities; for instance, VP4 upregulates IL-1α/β while suppressing TNF-α [88]. A distinct mechanism involves SVV 3Cpro, which functions as an atypical IL-1β-converting enzyme by directly cleaving and activating porcine pro-IL-1β in a species-specific manner, thereby circumventing the conventional inflammasome activation pathway [89].

#### 5.2.2. Antagonism of Host Innate Immunity by SVV Infection

**Figure 4:**
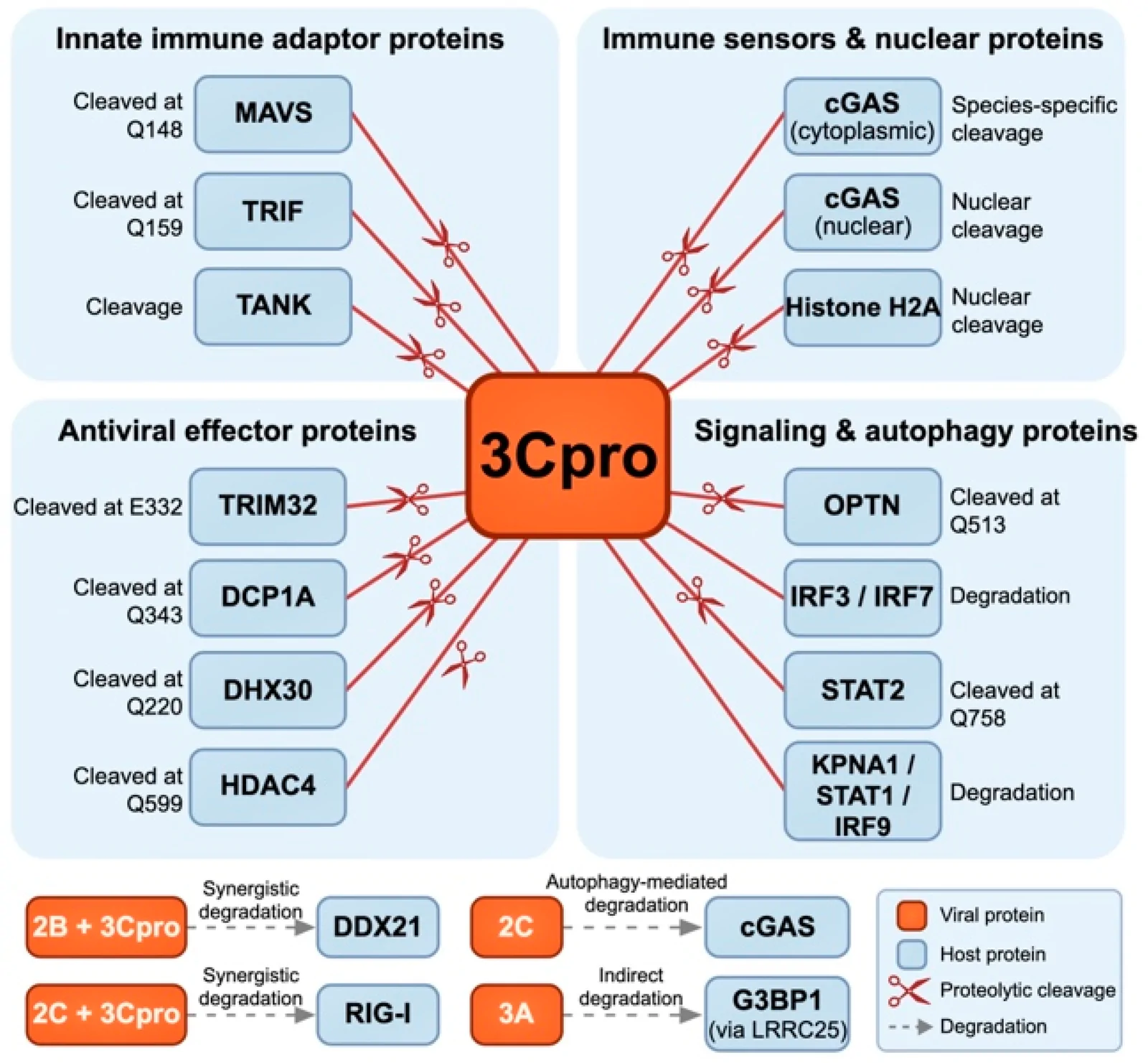
Cleavage of host proteins by SVV viral proteins (By Figdraw).

SVV has developed sophisticated strategies to counteract host innate immune responses, particularly targeting the type I interferon (IFN-I) signaling pathway, with the 3Cpro playing a pivotal role (Figure 4). The 3Cpro functions as a “molecular scissors,” inhibiting signal transduction by cleaving adaptor proteins across various signaling pathways. Specifically, it cleaves mitochondrial antiviral-signaling protein (MAVS) at glutamine 148 in the RLR pathway and TIR-domain-containing adapter-inducing interferon-β (TRIF) at glutamine 159 in the Toll-like receptor (TLR) pathway [72,90]. Additionally, 3Cpro exhibits species-specific cleavage of porcine cyclic GMP-AMP synthase (cGAS) [91], and can translocate into the nucleus to cleave nuclear cGAS and histone H2A [92]. Furthermore, 3Cpro targets intrinsic antiviral effector proteins of the host. It cleaves the E3 ubiquitin ligase TRIM32 at glutamic acid 332, thereby impairing its capacity to degrade VP3 and suppressing the IFN pathway [93]; cleaves the mRNA decapping enzyme DCP1A at glutamine 343, inhibiting its autophagy-mediated degradation of RNA-dependent RNA polymerase 3Dpol [94]; cleaves the RNA helicase DHX30 at glutamine 220, alleviating its restriction on viral replication [95]; and cleaves histone deacetylase HDAC4 at glutamine 599, preventing its degradation of 3Dpol and suppression of IFN signaling [96]. Moreover, the viral nonstructural proteins 2B and 3C collaborate to induce the degradation of another RNA helicase, DDX21 [97].

SVV employs multiple strategies to interfere with IFN-I signaling. Viral infection triggers reticulophagy by activating the PERK/ATF6 pathway, leading to the degradation of STING [66]. The 3C protease cleaves the autophagy receptor OPTN, thereby disrupting TBK1-IRF3 signaling [68]. Additionally, 3C protease exhibits deubiquitinase activity, inhibiting RIG-I, MDA5, TBK1, and TRAF3 by removing their ubiquitination [72,98]. The viral proteins 2C and 3C work together to promote RIG-I degradation [99], while 3A facilitates G3BP1 degradation by upregulating LRRC25 [100], and 2C induces cGAS degradation via autophagy [69]. Further downstream in the signaling pathway, 3C protease cleaves the TANK protein [90], and interacts with IRF3 and IRF7, inhibiting their phosphorylation and promoting their degradation [72]. To comprehensively inhibit antiviral responses, 3C protease cleaves STAT2 at Q758 and degrades STAT1, STAT2, and IRF9, as well as the nuclear import protein KPNA1, thereby disrupting the formation and nuclear translocation of the ISGF3 complex and attenuating ISG expression [101].

### 5.3. Adaptive Immune Response

SVV infection elicits rapid and robust adaptive immune responses. Regarding humoral immunity, elevated levels of virus-neutralizing (VN) antibodies become detectable within 5–7 days post-infection, with their titers exhibiting a significant negative correlation with the clearance of viremia and the resolution of clinical symptoms [56,60,102]. This early neutralizing activity is closely linked to the levels of IgM antibodies targeting the viral capsid proteins VP2 and VP3 [102]. Furthermore, increased levels of intestinal mucosal IgA and serum IgG are observed in pig breeds resistant to the disease [62]. Concerning cellular immunity, the infection also stimulates T-cell responses, characterized by an initial rise in the frequency of CD4^+^ T cells, followed by an increase in IFN-γ-producing CD8^+^ T cells [102]. Notably, substantial cross-protective immunity exists among different SVV strains, providing a theoretical foundation for vaccine development [57], and inactivated-virus vaccines have demonstrated the ability to induce effective protective immunity [103]. Nonetheless, SVV infection is detrimental to the immune system of neonatal piglets, leading to immunosuppression by inducing B-cell apoptosis. This phenomenon elucidates the inadequacy of adaptive immune responses in young animals to effectively control the infection [64].

## 6. Diagnosis, Prevention, and Control

### 6.1. Diagnostic Techniques

Etiological diagnosis is primarily employed to confirm acute infections, with real-time quantitative PCR (qRT-PCR) recognized as the “gold standard” for detection. Numerous highly sensitive and specific methodologies have been developed, utilizing SYBR Green I [104] and TaqMan probes [105]. For instance, a SYBR Green I qRT-PCR method targeting the VP1 region demonstrates high specificity and is capable of detecting SVV RNA across various sample types, including vesicular fluid, serum, and tissues, achieving an 88% detection rate in clinical samples and identifying transient viremia [104]. Infected pigs may shed the virus for up to 28 days, with viral RNA detectable in oral and nasal secretions as well as feces; the tonsils serve as the primary target tissue for virus isolation and detection [33]. Furthermore, qRT-PCR assays targeting the 3D polymerase gene are effective in detecting both historical and currently circulating strains [106]. Multiplex qRT-PCR assays, which can simultaneously detect and differentiate SVV from pathogens such as FMDV, are crucial for early outbreak response [107].

Various methods for on-site detection have been developed to enhance rapid diagnostic capabilities. A fluorescent-microsphere-based immunochromatographic test strip (FITS) enables the simultaneous detection of SVV and FMDV with a detection limit of 5 × 10^4^ PFU/mL [7]. Additionally, a field-deployable reverse transcription insulated isothermal PCR (RT-iiPCR) system, which utilizes recombinase polymerase amplification (RPA) to target the SVV-3D gene, offers a sensitivity of 7 copies/reaction and has been integrated into portable devices [108]. Furthermore, reverse transcription loop-mediated isothermal amplification (RT-LAMP), targeting the 5′ UTR and VP3-1 regions, provides another rapid and sensitive on-site detection method [109]. Additionally, the RPA-CRISPR/Cas12a technique, targeting the SVV-3D gene, exhibits a detection limit of 10 copies/μL and achieves 100% concordance with qRT-PCR, rendering it particularly suitable for resource-limited environments [110]. Similarly, an RT-RPA–CRISPR/Cas13a assay, which specifically detects SVV with no cross-reactivity against other porcine viruses, reaches a detection limit of 2.43 copies/μL and provides a result within 40 min, underscoring its suitability for on-site diagnostics [111]. Furthermore, the FIELD platform integrates room-temperature lysis with a one-step reaction, enabling the naked-eye detection of viral RNA at concentrations as low as 50 copies/μL within 35 min [112].

### 6.2. Prevention and Control Strategies

Inactivated vaccines currently represent the primary strategy in the development of SVV vaccines. Binary ethylenimine (BEI)-inactivated oil-adjuvanted vaccines have been shown to elicit high levels of neutralizing antibodies [113]. Studies involving animal challenges demonstrate that vaccination significantly reduces or mitigates clinical symptoms, viremia, and viral shedding, thereby offering substantial protection [12,113,114]. Additionally, maternal antibodies from vaccinated sows can confer passive immunity to piglets [103]. Moreover, a virus-like vesicle (VLV) vaccine, utilizing a VEEV replicon to express VP1 and VP2, induces robust humoral and cellular immune responses in pigs and provides effective protection. This approach offers a promising new direction for the development of safer and more efficient vaccines [4].

## 7. Conclusions and Future Perspectives

Taken together, this review provides a comprehensive overview of the virological properties, global epidemiological and evolutionary dynamics, molecular networks underlying virus-host interactions, and advances in diagnostic and control strategies for SVV. Current evidence has preliminarily elucidated the SVV replication cycle, immune evasion strategies, and pathogenic mechanisms, laying a critical theoretical foundation for the prevention and management of SVV-associated disease. Despite significant advancements in SVV research in recent years, numerous critical knowledge gaps persist. Notably, while different SVV strains exhibit considerable variability in pathogenicity, the molecular mechanisms underlying these differences remain inadequately understood. This lack of understanding hinders the accurate prediction of viral virulence evolution and the development of precise control measures. Secondly, SVV is capable of establishing a persistent infection in host animals, thereby serving as a potential reservoir for transmission. However, the mechanisms by which the virus circumvents host immune surveillance and clearance, as well as the specific molecular processes that sustain its persistent infection, remain to be fully elucidated. Additionally, the complete host range and natural transmission pathways of SVV have yet to be comprehensively defined.

Against this backdrop of outstanding knowledge gaps and an evolving evidence base, several limitations of this review should also be acknowledged. As a narrative review rather than a systematic review, the literature search and study selection were not governed by a formal PRISMA protocol, which may introduce selection bias in the studies discussed. To mitigate this potential bias within the scope of a narrative review, we conducted comprehensive literature surveys covering major peer-reviewed studies on SVV from its initial identification to the present, and cross-validated key conclusions against multiple independent research groups to ensure the reliability of the synthesis. For the mechanistic sections in particular, we prioritized original experimental studies conducted in porcine cells or live pigs that clearly delineated the viral protein mediator, the host target or signaling pathway, and the resulting functional outcome.

Building upon the identified knowledge gaps and contextual limitations, future research on SVV should prioritize three critical areas.

Firstly, the development of more effective broad-spectrum vaccines and antiviral drugs remains an essential priority for controlling SVV transmission within swine populations. Although substantial cross-protective immunity has been observed across SVV strains and the conserved neutralizing epitopes on VP2 provide a strong rationale for a broad-spectrum vaccine strategy, direct evidence of cross-lineage protection remains limited and requires further validation. This endeavor necessitates a comprehensive analysis of the mechanisms underlying virus-host immune interactions. Based on this foundation, a key research objective is to ascertain whether a multivalent vaccine incorporating these conserved neutralizing epitopes offers superior cross-lineage protection compared to conventional single-strain vaccines.

Secondly, given the persistent emergence of variant SVV strains, enhanced surveillance capacity and deeper mechanistic understanding constitute two complementary priorities. On one hand, it is imperative to develop tools that enable prompt identification of new strains, with particular emphasis on field-deployable on-site detection capabilities. On the other hand, the molecular determinants of viral pathogenicity and persistent infection remain largely uncharacterized. Specifically, reciprocal mutagenesis approaches can be utilized to assess whether specific residues within the 3C protease are responsible for the differential virulence across SVV lineages. In addition, a critical outstanding question is whether disrupting the viral proteins that sustain tonsillar persistence will abrogate the carrier state and reduce onward transmission.

Lastly, given SVV’s inherent oncolytic properties, ongoing research into its interactions with host organisms will provide valuable insights for both viral infection biology and cancer research.

## Figures and Tables

**Table 1:** Modulation of host cellular processes by SVV.

Cellular Process	Viral Mediator	Host Target/Pathway	Effect	Reference
Autophagy	Intact viral infection	Complete autophagic flux	Species-specific: enhances viral proliferation in porcine cells, suppresses it in human cell lines	[65]
	Intact viral infection	Autophagosome; PERK and ATF6 arms of UPR	Autophagosomes serve as sites of viral replication; autophagy initiation upon ER stress	[32]
	3Cpro	STING	Degradation via reticulophagy, facilitating immune evasion	[66]
	VP1, VP3, 3C	AKT–AMPK–MAPK/mTOR axis	Induction of autophagy	[67]
	3Cpro; VP1	OPTN (Q513); SQSTM1/p62 (E355, Q392, Q395)	Cleavage of selective autophagy receptors abrogates their antiviral activity; VP1–p62 interaction promotes p62 degradation	[65,67,68]
	Intact viral infection	cGAS; EphA2	Autophagic degradation of cGAS; EphA2 cleavage disrupts mTOR signaling	[69,70]
Apoptosis	3Cpro	Caspase-3, -8, -9	Late-stage activation of apoptotic signaling, promoting viral release and dissemination	[71,72]
	Intact viral infection	ER stress–PERK; mitochondrial Ca^2+^	Mitochondrial Ca^2+^ overload activates the mitochondrial apoptotic pathway	[31]
Pyroptosis	3Cpro	GSDMD (Q193, Q277)	Direct cleavage induces pyroptosis	[72,73]
	3Cpro	GSDMA (Q187–G188)	Cleavage suppresses GSDMA-mediated pyroptosis	[74]
	3Cpro, 3Dpol	NLRP3 (Q305)	3Cpro cleavage inhibits inflammasome activation, whereas 3Dpol activates the NLRP3 inflammasome, indicating antagonistic regulation	[72,75,76]
Ferroptosis	3Cpro, 3Dpol	GPX4; NCOA4-mediated ferritinophagy	GPX4 degradation and ferritinophagy drive lipid peroxide accumulation, triggering ferroptosis	[77]
DNA damage response	2B, 2C	53BP1	DDR activation with impaired DNA repair (“damage without repair”)	[24]
Translation	3Cpro	PABPC1	Shutoff of host protein synthesis	[78]
	Intact viral infection	RPL13; viral IRES	RPL13 recruitment enhances IRES-driven viral translation	[18]
Stress granules	3Cpro	PKR–eIF2α; G3BP1–eIF4G1	Early transient SG induction; late SG disassembly via G3BP1–eIF4G1 disruption or eIF4G1 cleavage	[72,79]
Metabolism	Intact viral infection	SLC38A8	Aspartate elevation facilitates viral replication	[80]
Copper homeostasis	Intact viral infection	Copper; NF-κB	Copper accumulation inhibits NF-κB signaling, attenuating innate immune responses	[81]

## Data Availability

No new data were created or analyzed in this study. Data sharing is not applicable to this article.

## Abbreviations

The following abbreviations are used in this manuscript:

SVV	Seneca Valley virus
SVA	Senecavirus A
PIVD	porcine idiopathic vesicular disease
FMD	foot-and-mouth disease
ORF	open reading frame
UTR	untranslated region
IRES	internal ribosome entry site
HCV	hepatitis C virus
CSFV	Classical swine fever virus
3Cpro	3C protease
L	leader protein
DDR	DNA damage response
3Dpol	3D polymerase
RdRp	RNA-dependent RNA polymerase
ANTXR1	anthrax toxin receptor 1
TEM8	tumor endothelial marker 8
ER	endoplasmic reticulum
SNV	single-nucleotide variant
UPR	unfolded protein response
GSDMD	gasdermin D
GSDMA	gasdermin A
DSB	double-strand break
SG	stress granule
RLR	retinoic acid-inducible gene I-like receptor
mtDNA	mitochondrial DNA
IFN-I	type I interferon
MAVS	mitochondrial antiviral-signaling protein
TRIF	TIR-domain-containing adapter-inducing interferon-β
TLR	Toll-like receptor
cGAS	cyclic GMP-AMP synthase
VN	virus-neutralizing
qRT-PCR	real-time quantitative PCR
FITS	fluorescent-microsphere-based immunochromatographic test strip
RT-iiPCR	reverse transcription insulated isothermal PCR
RPA	recombinase polymerase amplification
RT-LAMP	reverse transcription loop-mediated isothermal amplification
BEI	Binary ethylenimine
VLV	virus-like vesicle
